# Upper Gastrointestinal Bleeding as a Debut Form of Groove Pancreatitis: A Diagnostic Challenge

**DOI:** 10.1155/2022/5562778

**Published:** 2022-03-07

**Authors:** Belén Matías-García, Camilo José Castellón-Pavón, Gustavo Díaz-García, Belén Manso-Abajo, Daniel Hernández-Aceituno, Antonio Hermosín-Peña, Luis Mejías-Sosa, Manuel Durán-Poveda

**Affiliations:** ^1^General and Digestive Surgery Department of Príncipe de Asturias Teaching Hospital, Alcalá de Henares, Madrid, Spain; ^2^General and Digestive Surgery Department of Rey Juan Carlos Teaching Hospital, Móstoles, Madrid, Spain; ^3^Radiology Department of Rey Juan Carlos Teaching Hospital, Móstoles, Madrid, Spain; ^4^Interventional Radiology Department of Rey Juan Carlos Teaching Hospital, Móstoles, Madrid, Spain; ^5^Pathology Department of Rey Juan Carlos Teaching Hospital, Móstoles, Madrid, Spain

## Abstract

**Introduction:**

Groove pancreatitis (GP) is an unusual subtype of chronic pancreatitis that affects the groove area. Differential diagnosis between groove pancreatitis and pancreatic carcinoma (PC) can be challenging, both clinically and radiologically. Our aim is to report the first case of GP debut with upper gastrointestinal bleeding (UGB). *Case Report*. A 53-year-old man with a personal history of alcohol and tobacco abuse and chronic pancreatitis was admitted to the hospital for epigastric abdominal pain. A computed tomography scan showed a locally advanced neoformative lesion in the distal stomach. The patient presented melena, arterial hypotension, and 4.4 g/dl of hemoglobin. An upper gastrointestinal endoscopy showed a neoformative ulcerated lesion at the duodenal bulb without active bleeding. Biopsies were taken, and histopathological analysis did not show malignancy. A cephalic pancreaticoduodenectomy was performed, and the postoperative period was uneventful. Histopathological analysis revealed a segmental GP. *Discussion*. GP is an uncommon entity, and its clinical and radiological presentation mimics PC. However, with advances in imaging tests, several radiological criteria have been described to distinguish GP from PC preoperatively. Although some authors recommend a stepwise management with initial conservative therapy, a therapeutic strategy has not yet been established.

**Conclusion:**

GP is an uncommon type of focal pancreatitis that should be considered as a differential diagnosis of PC. We report the first clinical case of GP whose debut with UGB presented a greater diagnostic and therapeutic challenge.

## 1. Introduction

This entity was first described by Becker in 1973 [1]. However, it was not until 1982 that Stolte et al. adopted the name groove pancreatitis (GP) [2]. GP is an unusual subtype of chronic focal pancreatitis that affects the pancreatoduodenal groove. The pancreatoduodenal groove is the area bordered by the vessels in the second portion of the duodenum (laterally), head of the pancreas and the common bile duct (medially), the first portion of the duodenum and gastric antrum (anteriorly), and the third portion of the duodenum or inferior vena cava (posteriorly) [3]. In the literature, different names have been used to refer to the same entity such as paraduodenal pancreatitis, cystic dystrophy of the duodenal wall, pancreatic hamartoma of the duodenum, myoadenomatosis, and duodenal cystic dystrophy in heterotopic pancreas, but the term groove pancreatitis has established its place as the name for this disease [4, 5]. In 1991, Becker et al. [1] categorized GP in the pure form, which involves the groove exclusively and does not involve the main pancreatic duct, and the segmental form, which involves the groove and the head of the pancreas that can lead to obstruction of the main pancreatic duct [1, 6]. Either one of these two forms can be solid or cystic [4]. The prevalence varies from 2.7 to 24.5% of patients who underwent pancreatic resection for chronic pancreatitis [3, 7]. It is more common in men in his fourth or fifth decade of life [8], and its etiology seems to be mainly related to alcohol and tobacco abuse [7, 8]. However, its pathogenesis is not clearly established. The initial presentation usually includes nonspecific symptoms such as upper abdominal pain, nausea, and vomiting [8]. This unspecific clinical manifestation can lead us to erroneously suspect pancreatic carcinoma (PC). Due to its low incidence and its clinical similarity to PC, preoperative diagnosis becomes challenging. This study is aimed at reporting the first case of GP debut with upper gastrointestinal bleeding (UGB).

## 2. Case Report

**Figure 1:**
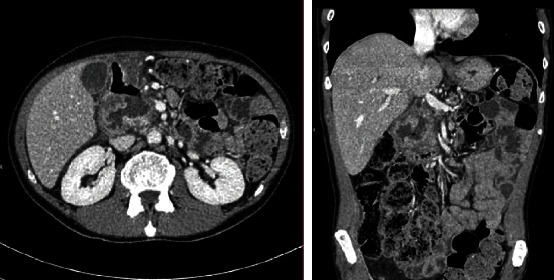
Computed tomography findings: parietal thickening affecting the antropyloric region and first duodenal portion with an ulcerated appearance as well as trabeculation of the adjacent fat and the presence of adenopathies, initially interpreted as suspicious of a neoformative nature.

**Figure 2:**
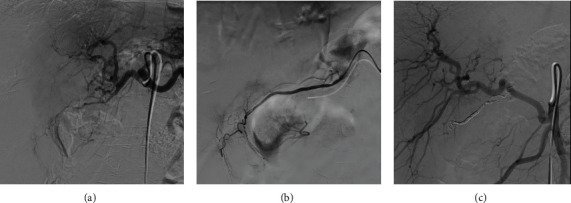
(a) Arteriography of the celiac trunk prior to embolization. (b) Selective arteriography of the gastroduodenal artery prior to embolization. (c) Arteriography after gastroduodenal artery embolization.

**Figure 3:**
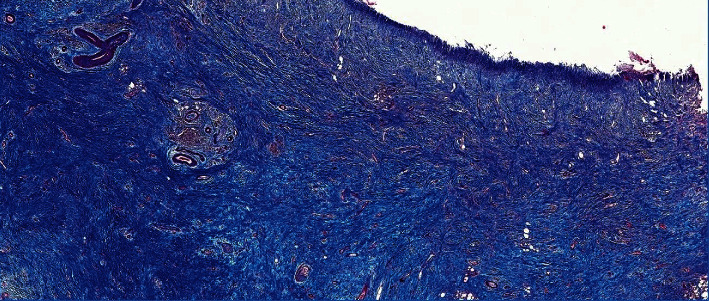
Trichrome stain (Masson); 2x: pancreas with extensive fibrosis.

A 53-year-old man with a personal history of alcohol and tobacco abuse, arterial hypertension, diabetes mellitus, and pancreatitis of alcoholic origin consulted in the emergency department with epigastric abdominal pain, not irradiated, associated with nausea and vomiting. The analysis highlights 15.9 × 10^3^*μ*l leukocytes (87.4% neutrophils), 9.4 g/dl of hemoglobin, 736 × 10^3^*μ*l platelets, 0.26 mg/dl of total bilirubin, C-reactive protein 0.77 mg/dl, and normal hepatopancreatic profile. The requested tumor markers (carcinoembryonic antigen and CA19.9) were normal. An abdominal ultrasound was performed, showing cholelithiasis and dilation of the extrahepatic bile duct. Subsequently, a computed tomography (CT) scan was performed which we interpreted as a locally advanced neoformative lesion in the distal stomach, which involved lymphatic nodes and gastroduodenal artery and invaded the pancreatic head, with dilatation of the distal common bile duct (Figure 1). Subsequently, the patient presented melena, arterial hypotension, and 4.4 g/dl of hemoglobin. The upper gastrointestinal endoscopy performed showed an ulcerated lesion at the duodenal bulb, with raised edges and a fibrin base, without evidence of active bleeding in this moment. Biopsies were taken, and histopathological analysis did not show evidence of malignancy. Nevertheless, a selective embolization of the gastroduodenal artery was performed with the intention of preventing new bleeding and completing the study (Figure 2). Nuclear magnetic resonance imaging (MRI) was performed, which we interpreted as a locally advanced antroduodenal neoformative process. Given the clinical persistence of abdominal pain that was difficult to control with analgesia and the suspicion of malignancy, it was decided to perform an exploratory laparotomy. A resectable mass was visualized that encompassed the pancreatic head, duodenum, and antrum. Distant disease was not observed, and a cephalic pancreaticoduodenectomy (CPD) was performed. The postoperative period was uneventful, and the patient was discharged on the 7th postoperative day. Histopathological analysis revealed a loss of acini and ductal tissue, with relative sparing of islets, dilated ducts, chronic inflammation, and extensive fibrosis with extension to the duodenal wall (Figure 3). No infiltrative neoplasia was observed.

## 3. Discussion

GP is an uncommon entity, and its prevalence varies from 2.7 to 24.5% in surgical specimens of pancreaticoduodenectomies performed in patients with chronic pancreatitis [3, 7]. Its pathogenesis is not clearly established [6]. However, it is reported that the chronic abuse of alcohol and tobacco is the factor mainly related to this entity due to the increase in the viscosity of the pancreatic juices, causing stasis and obstruction of the outflow in pancreatic ducts [2, 3, 7]. This causes an increase in pressure within the Santorini duct and the consequent release of secretion in the groove [5, 9]. This leads to a hyperplasia of the Brunner's glands, which is the most common finding in histological analysis [5] and causes occlusion or dysfunction of the minor papilla [6–8]. Other causes of minor papilla dysfunction that are related to this entity are a history of gastrectomy, gastroduodenal ulcer, and biliary diseases and the presence of anatomic abnormalities such as ectopic pancreatic tissue in the duodenum or pancreatic divisum [10, 11]. The initial clinical presentation is nonspecific, and the most common manifestations are upper abdominal pain, nausea, vomiting, and weight loss, primarily due to duodenal obstruction [5, 8, 12]. Diarrhea or diabetes mellitus have also been reported to be commonly associated with GP [5]. The patient in our case had a personal history of alcohol and tobacco abuse and clinical symptoms similar to those present in GP, except those suggestive of UGB since there is no reported case of GP that debuted with gastrointestinal bleeding. However, it is reported in the literature that gastroduodenal ulcer can be a cause of GP [9, 13], in addition to gastrointestinal bleeding. Therefore, in our case, we cannot clarify whether the cause of GP is related to a personal history of alcohol and tobacco abuse, to gastroduodenal ulcer, or both. In laboratory findings, pancreatic and liver enzymes may be elevated in the acute period [8], as well as tumor markers such as carcinoembryonic antigen and CA19.9 that can be elevated in up to a third of patients [4]. Some studies have reported that CA19.9 levels are significantly higher in PC than in GP [4]. However, it has not yet been possible to establish a cutoff value for the differentiation of these two entities [4]. Laboratory tests and tumor markers in our patient were normal in the acute period.

Given its low incidence and nonspecific symptoms, GP poses a diagnostic challenge. Additionally, inflammation and fibrosis can result in a pseudotumor that mimics PC on diagnostic imaging tests [8]. Despite the advances in high-quality imaging techniques, distinguishing this entity from PC is difficult and about 60% are misdiagnosed preoperatively as cancer as in our patient [8]. However, three MRI criteria have been described with the aim of preoperatively distinguishing GP from PC: focal duodenal thickening, contrast enhancement of the second portion of the duodenum, and cystic lesions of the accessory pancreatic duct [13, 14]. These findings support the diagnosis of GP over PC with an accuracy of 87.2% and negative predictive value for cancer of 92.2% [5]. Other findings present on MRI may be a sheet-shaped mass located at the level of the groove, which is hypointense on T1-weighted images and iso- or slightly hyperintense on T2-weighted images according to the time of disease [5, 7]. On CT scan, the finding in the pure form of the disease is a hypodense lesion as a curvilinear crescentic shape between the pancreatic head and the duodenum [5, 6]. This lesion may show a delayed enhancement of contrast on both CT and MRI that reflects its fibrous nature [6, 7]. Additional findings include duodenal stenosis and cystic lesions in the groove area [6]. In the pure form of the disease, the pancreatic duct usually appears normal [6]. In the segmental form, the pancreatic head is also affected, which can result in a dilation of the pancreatic duct [6]. Today, endoscopic ultrasound (EUS) is also important in the diagnosis of GP. EUS allows to detect thickening and stenosis of the second duodenal portion, intramural cysts, and smooth tubular stenosis of the common bile duct without abnormality of the main pancreatic duct in the pure form [5, 7]. In the segmental form, EUS can show a heterogeneous and hypoechoic mass, enlargement of the pancreatic head, with calcifications or pseudocyst and dilatation of the main pancreatic duct [5]. In addition, EUS allows us to take biopsies, which is why it has become the most appropriate diagnostic test to differentiate between GP and PC [5]. However, when the tissue sample obtained by EUS is positive for PC, it can be definitive, but when it is negative, the possibility of sampling error should always be considered [9]. In the case of our patient, the histological analysis of the samples was negative for malignancy. However, given the unusual clinical presentation with UGB, this result was considered a possible error and management was performed in relation to a possible PC. Finally, a recent report suggests that FDG-PET-CT may be useful as a diagnostic imaging test by demonstrating multiple areas of FDG in the paraduodenal tissues as opposed to a single confluent mass in the head of the pancreas as what occurs with PC [9, 15].

Although some authors recommend stepwise management with initial conservative therapy [3, 4], a therapeutic strategy has not yet been established and treatments include medical, endoscopic, or surgical approaches. Conservative treatment consists of cessation of alcohol and tobacco abuse, pancreatic rest, analgesics, proton pump inhibitors, pancreatic enzyme supplement, and nutritional support [3, 7]. Endoscopic treatment consists of drainage of the stenotic or obstructed pancreatic duct, which reports good results in the literature [7, 16]. In a systematic review that included 335 GP, complete symptom relief was observed in 50% of patients who were treated conservatively and 57% who underwent endoscopic treatment [16]. However, in the event of nonsurgical treatment failure, the presence of complications, or the suspicion of a neoplasm, surgical treatment is of choice [7, 8]. Cephalic duodenopancreatectomy is the preferred surgical technique because it allows the control of symptoms, establishes the definitive diagnosis, and prevents recurrence [7, 8]. Complete relief of abdominal pain in 76-79% of patients undergoing surgical treatment is reported in the literature [16, 17]. In our case, due to the persistence of the symptoms, the antecedent of UGB, and the persistent suspicion of neoplasia, we decided on the initial surgical treatment.

## 4. Conclusion

GP is an uncommon type of focal pancreatitis whose clinical and radiological diagnosis is challenging. Its management is not clearly established, although some authors recommend a stepped management beginning with conservative treatment. However, given the difficulty in obtaining a definitive diagnosis or, as occurred in our case, in the presence of complications, surgical resection is often required. We report the first clinical case of GP whose debut with UGB presented a greater diagnostic and therapeutic challenge.
